# Serological and Molecular Evidence of Bovine Leukemia Virus in Sheep Populations in the South of Iran

**DOI:** 10.1002/vms3.70303

**Published:** 2025-04-02

**Authors:** Azadeh Yektaseresht, Mohsen Ghane, Rozhin Kargar, Mohammad‐Sadegh Golvajouei

**Affiliations:** ^1^ Department of Pathobiology School of Veterinary Medicine Shiraz University Shiraz Iran; ^2^ Department of Clinical Sciences School of veterinary medicine Shiraz University Shiraz Iran; ^3^ Biotechnology Center School of Veterinary Medicine Shiraz University Shiraz Iran

**Keywords:** Bovine leukemia virus (BLV), ELISA, Nested PCR, Sheep, Southern Iran

## Abstract

Bovine leukemia virus (BLV) is a neoplastic lymphosarcoma affecting cattle worldwide. Sheep, as one of the multiple host species for BLV, can transmit the virus to various animals and potentially to humans. Recognizing a gap in research, particularly in southern Iran, where the prevalence of BLV in sheep populations has not been investigated, this study aimed to fill this gap. One hundred blood samples were collected from seven flocks of sheep in the Fars province, Shiraz, southern Iran. Our study specifically investigated the prevalence of BLV in sheep populations in southern Iran, using serological and molecular analyses. Anti‐gp51 IgG antibodies and proviral DNA were detected using ELISA and nested PCR, respectively. In this study, 37% of the 100 sheep tested positive for BLV IgG antibodies from the Fars province in southern Iran. The gag BLV gene with a band length of 385 bp was detected in 3% (3/100) of the samples. In this study, we examined the evidence of BLV in sheep from southern Iran. Control measures are needed to reduce infection in definitive hosts.

## Graphical abstract

•Sheep are one of several host species for bovine leukemia virus which can generate several public health concerns, such as potential transmission and spread of zoonotic pathogens to individuals.

•This is the first report of bovine leukemia virus serological and molecular status in sheep.

•The results of this study showed the presence BLV among sheep in southern Iran, which could be a public health concern. According to the prevalence of BLV infection in sheep, implementation of control measures to reduce infection in definitive hosts are needed.

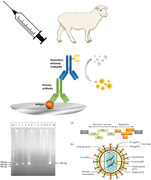

## 1 Introduction

Bovine leukemia virus (BLV) causes lymphosarcoma in cattle (Gillet et al. 2007; Polat et al. 2017). Approximately 70% of cattle with BLV infection show no symptoms, indicating a subclinical infection. Persistent lymphocytosis (PL), regarded as a benign form of leukemia, may affect approximately one‐third of infected cattle. Approximately 1%–5% of these may promote malignant B‐cell lymphoma tumours in vital organs, potentially causing several functional abnormalities incompatible with survival (Gillet et al. 2007; Marawan et al. 2021). BLV also infects various species, including sheep, water buffalo, and alpacas (Buehring et al. 2019). The infection with BLV in sheep causes B‐cell lymphoma and leukemia (Gillet et al. 2007; Mousavi et al. 2014; Porta et al. 2019). As explained earlier, in addition to cattle, sheep are natural host of BLV. Small dairy ruminants, primarily found in the subtropical‐temperate regions of Asia, Europe, and Africa, contribute to ∼3.5% of worldwide milk production. In addition, out of the annual mean consumption of 41.6 kg meat per person, the global consumption of sheep meat in 2017 was ∼2.5 kg per person (Pulina et al. 2018). First identified in 1871, this disease lacks any known treatment and causes economic losses in the cattle industry. BLV decreases milk production, weight loss, and abortion risk and causes other adverse clinical effects. It may also indirectly lead to import restrictions on animals from BLV‐affected regions. Owing to these factors, enzootic bovine leukosis (EBL) has the potential to significantly affect global trade (Marawan et al. 2021; Polat et al. 2017). BLV belongs to the Retroviridae family that includes enveloped viruses that stand apart from other viruses because of their distinct replication cycle. At least four genes encode the structural and replication proteins of an infectious virus, and are arranged in the following order: 5’‐gag‐pro‐pol‐env‐3’. The gag gene (which we wanted to detect in this study) encoded viral structural proteins are initially translated as a precursor to the gag polyprotein (Hunter 2008). Two common approaches are employed to detect BLV: serological methods and polymerase chain reaction (PCR) (Bartlett et al. 2020). For serological testing, the OIE (Office International des Epizooties) endorses enzyme‐linked immunosorbent assay (ELISA) and agar‐gel immunodiffusion (AGID) as reference methods for detecting antibodies targeting BLV gp51 and p24 proteins. A range of PCR techniques exist, each offering distinct advantages. Sensitivity levels are significantly higher with semi‐nested and nested tests than with single PCR, which is why they are used more frequently to detect BLV (Marawan et al. 2021). Crossing the species barrier also increases the risk of spillover to humans (Sanchez et al. 2021). Evidence has shown virus presence in humans and other livestock species, and potential links between BLV and human carcinoma may suggest its zoonotic character (Khan et al. 2022; Olaya‐Galan et al. 2022). Nucleotide sequencing has enabled researchers to demonstrate that the BLV DNA found in breast tissue is genetically similar to that of the bovine virus. However, it is unclear whether and how BLV is transmitted to humans (de Quadros et al. 2023). One of the most significant concerns regarding these infections in multiple animal species is that certain animals may remain asymptomatic, potentially serving as intermediate hosts and playing a crucial role in the dissemination of pathogens (Brooks et al. 2021). The objective of this study was to determine whether the virus is circulating in sheep within the study area, which would provide insights into the control programs for BLV in the region.

## 2 Methodology

### 2.1 Study Site

This study was performed in the Fars province, Shiraz, southern Iran, located at 29°37′ N‐52°32′ E.

### 2.2 Sampling

Between February 2023 and January 2024, researchers gathered 100 blood samples from seven flocks of Iranian sheep, (≤3 years old). The study focused on animals from traditional farms, selected at random. It is worth noting that throughout the study period and in previous farm records, no clinical symptoms or lymphosarcoma were detected. For serological testing, serum samples were extracted and kept at −20°C. For molecular analysis, blood samples were preserved in EDTA and vacutainer clot activator tubes.

### 2.3 Enzyme‐Linked Immunosorbent Assay

Sera were analyzed for BLV gp51 antibody utilizing a commercial ELISA kit (SVANOVA Indirect ELISA). Positive and negative sheep sera were used as controls. The ELISA test was validated by ensuring that the positive control reacted with the gp51 BLV antigen in the kit and that the negative control did not produce a specific reaction with the gp51BLV antigen. Sheep serum samples were incubated with horseradish peroxidase anti‐sheep IgG conjugate (Sigma, USA) diluted 1:5000. The assay was conducted and analyzed according to the modified manufacturer's instructions. Samples were classified as negative if their optical density (OD) value was less than 0.25, and positive if the OD value exceeded 0.25.

### 2.4 Polymerase Chain Reaction

Using a DNA extraction kit (Cinacolon, Tehran, Iran), genomic DNA was isolated from whole blood cells. Nested PCR was employed to analyze all DNA samples for a 385‐bp segment of the gag gene. A sample extracted using BLV (kindly provided by Professor Gholamreza Nikbakht Brujeni) served as the positive control, while a sample without the target sequence was used as the negative control. Two pairs of primers, as proposed by Wang et al. (2002), were employed for polymerization. PCR was conducted using a total volume of 20 µL. This reaction mixture comprised 10 µL of Amplicon's Taq DNA Polymerase 2× Master Mix, 5 µL of distilled water, 1 µL of each primer at a concentration of 10 µM, and 3 µL of DNA template. The initial PCR cycle began with denaturation at 94°C for 3 min, then proceeded through 30 cycles consisting of denaturation (94°C, 60 s), annealing (55°C, 60 s), and extension (72°C, 120 s), concluding with a final extension at 72°C for 10 min. The subsequent PCR round started with denaturation at 94°C for 3 min, followed by 30 cycles of denaturation (94°C, 45 s), annealing (59°C, 45 s), and extension (72°C, 40 s), ending with a final extension at 72°C for 5 min. Following electrophoresis of PCR products on 1.5% agarose gel, images were obtained using the LabNet gel documentation system.

### 2.5 Data Analysis

All analyses were performed using the SPSS software, version 16.0 (SPSS Inc., Chicago). Significance was defined at *p* value < 0.05 (Table 1)

**TABLE 1:** The analysis covered a number of sheep from different flocks in the Fars province, Shiraz, situated in southern Iran.

Flock	Number of sheep analyzed^a^	Number of positive sheep molecularly	Number of seropositive sheep
A	20	—	11
B	17	—	6
C	20	3	12
D	11	—	3
E	8	—	—
F	13	—	3
G	11	—	2
Total	100	3	37

^a^ Blood and serum samples were taken from female sheep, aged between 2 and 6 years, in the herds of the Fars province, Shiraz, southern Iran, as mentioned in this table.

## 3 Results

**FIGURE 1:**
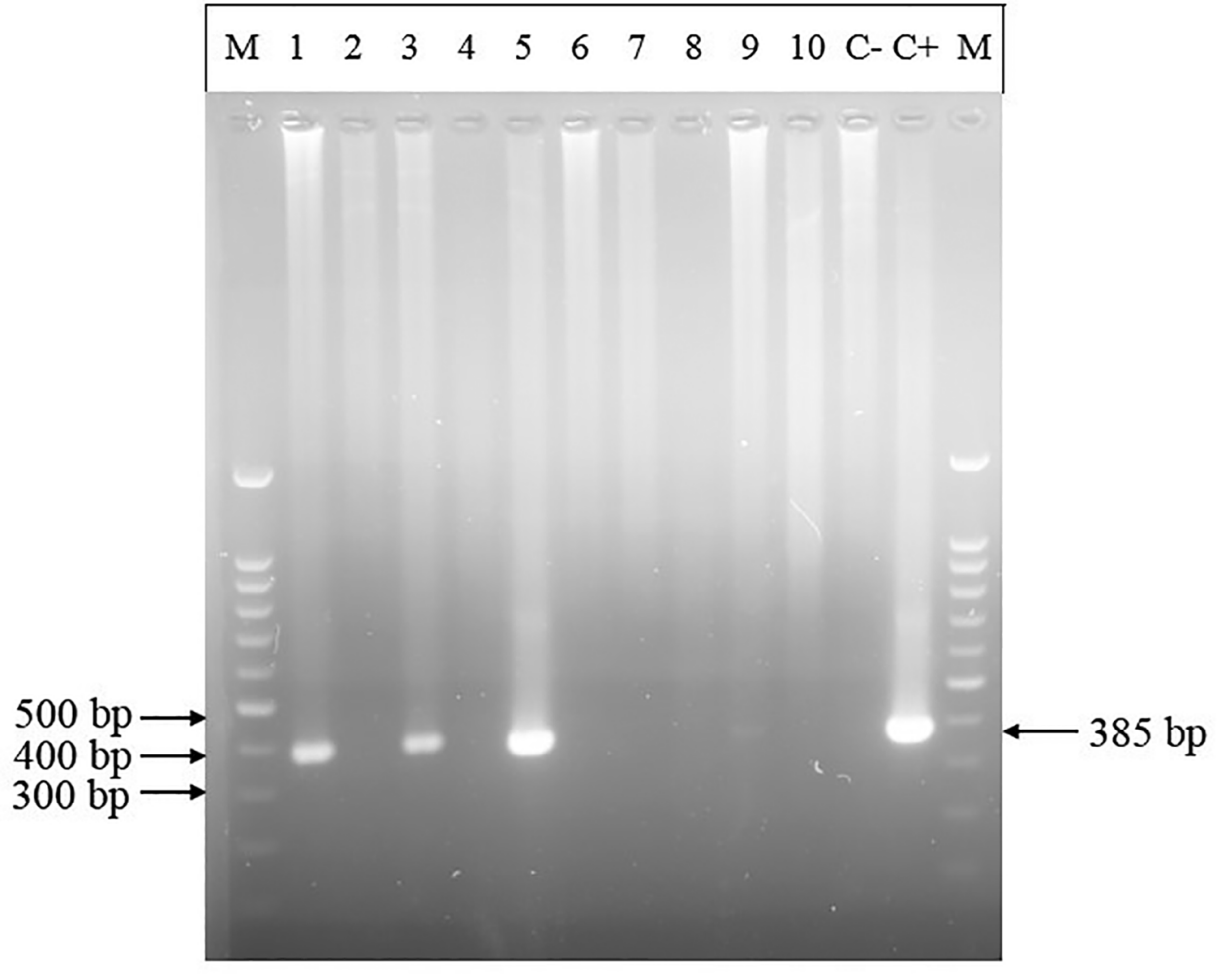
Agarose gel illustrating the electrophoretic resolution of amplified products of *gag* gene of Bovine Leukemia virus (BLV). Lane M: 100 kb ladder; lanes 1, 3 and 5: samples carrying BLV infection or samples in which DNA fragments of 385 bp were amplified using primers targeting the *gag* gene; lanes 2, 4, 6, 7, 8, 9, and 10: samples negative for BLV infection or samples in which primers targeting *gag* gene did not amply specific DNA fragment of BLV; lane C−: negative controls in which distilled water was used instead of DNA; lane C+: positive control in which purified BLV DNA was used as template.

Serological testing utilizing ELISA revealed that 37 of the 100 (37%) serum samples exhibited positive results for anti‐BLV antibodies (Table 1). A total of 100 whole blood samples were analyzed for the presence of BLV via PCR. Three of the 100 (3%) samples demonstrated positive results using nested PCR (Table 1), yielding a band of the expected size (385 bp) (Figure 1).

## 4 Discussion

BLV is widespread throughout the world. The diagnosis of BLV is essential to prevent its spread. The detection of BLV in multiple species increases the challenge of management and control of viral infection and monitoring its dissemination and transmission patterns. The lack of infection control in sheep and cattle facilitates the dissemination of BLV through livestock trading. This study provides a new serological and molecular evidence for BLV in Iranian sheep. In this study, we monitored BLV infection in sheep by the detecting of both viral‐specific antibodies and proviral DNA. This is the first study to investigate evidence of BLV among sheep populations in southern Iran. Several studies from different countries have confirmed the presence of BLV in sheep. A study conducted in Colombia examined 44 sheep and 61 buffaloes using nested PCR. The results revealed BLV presence in 25.7% of the animals, specifically 12 buffaloes and 15 sheep. Within this investigation, the proportion of sheep infected with BLV was determined to be 14.28% (Olaya‐Galan et al. 2022). Nekoei et al. (2015) reported a 5.3% The prevalence rate of 5.3% has been reported in sheep from Central and Southwest of Iran (Nekoei et al. 2015). The study revealed that three of 100 sheep (3%) had molecular evidence of BLV exposure. Our results were comparable to those reported in Central and Southwest of Iran (5.3%) (Nekoei et al. 2015). Animals infected with BLV develop immunity against the envelope protein env gp51, detectable serologically through immune diffusion tests and ELISA (Mohammadabadi et al. 2011). ELISA is a laboratory method with very high sensitivity. The ELISA is currently considered more sensitive than alternative serological assays, like the AGID test (Radostits et al. 2007). Researchers have used ELISA to control the virus at the herd level because of its high sensitivity compared to AGID (Radostits et al. 2007; Smith 2002; Tajima and Aida 2002). Limited serological research has been conducted globally to identify BLV in sheep populations. A study conducted by del Fava (2010) in Brazil detected BLV antibodies in sheep at a rate of 0.07% using the agar‐gel immunodiffusion technique. In this study 37 of 100 sheep (37%) had serological evidence of BLV exposure. Our results demonstrate that the infected animals were seropositive, which possibly depended on whether the sheep had stable lymphocytosis and whether malignant lymphosarcoma tumours had developed (Radostits et al. 2007). The results of this study showed that the AP3D1 receptor of BLV, a transporter protein present in mammals (Odorizzi et al. 1998), was able to bind to the gp51 protein of BLV. This protein shows a high degree of conservation in cattle and sheep. Various studies have investigated the relationship between breast cancer and BLV. Researchers have conducted serological studies, testing serum samples from cancer patients and individuals potentially exposed to BLV to identify BLV antibodies (Buehring et al. 2019; Onuma et al. 1987; Slavikova et al. 1987). Therefore, BLV plays a significant role in the initiation of cancer in human tissues (Khan et al. 2022). The results of this study suggest that BLV infects other species, and few strategies are available for its prevention and control.

## 5 Conclusion

BLV is circulating in the sheep population of Fars province, highlighting the role of sheep as a reservoir host in the epidemiology of this viral disease. Contact between sheep and cattle could provide additional opportunities for further onward transmission of the virus. The presence of BLV in sheep suggests that BLV can infect multiple species and should be taken t the prevention programs for effective epidemiological surveillance.

## Author Contributions

Azadeh Yektaseresht: supervision, methodology, validation, visualization, investigation, data curation, writing–original draft, writing–review and editing. Mohsen Ghane: methodology, investigation, writing–review and editing, resources, conceptualization. Rozhin Kargar: methodology, validation, visualization, data curation, writing–review and editing. Mohammad‐Sadegh Golvajouei: methodology, validation, visualization, data curation, writing–review and editing.

## Ethics Statement

This study was approved by the research committee of the Faculty of Veterinary Medicine,Shiraz University, Shiraz, Iran and documented by the number: 983/46/22. The authors confirm that the ethical policies of the journal, as noted on the journal's author guidelines page, in the Faculty of Veterinary Medicine, Shiraz University, Iran (letter No. 983/46/22). All methods were carried out in accordance with Shiraz University animal welfare guidelines and policies.

## Conflicts of Interest

The authors report no conflicts of interest.

### Peer Review

The peer review history for this article is available at https://publons.com/publon/10.1002/vms3.70303.

## Data Availability

The data that support the findings of this study are available on request from the corresponding author.
